# Predicting residue ionization of OmpF channel using Constant pH Molecular Dynamics as benchmark

**DOI:** 10.1371/journal.pcbi.1013628

**Published:** 2025-10-23

**Authors:** Ernesto Tavares-Neto, Marcel Aguilella-Arzo, Vicente M. Aguilella

**Affiliations:** Laboratory of Molecular Biophysics, Department of Physics, Universitat Jaume I, Castellon, Spain; Uppsala Universitet, SWEDEN

## Abstract

Electrostatic interactions are crucial for protein structure and function, especially in mesoscopic protein channels where ion selectivity is largely governed by the protein’s electrostatic properties. Understanding the protonation state of ionizable residues across pH values —often described by their p*K*_a_— is key to linking structure and function. However, experimental p*K*_a_ determination is challenging, typically carried out using Nuclear Magnetic Resonance only in a limited number of membrane proteins. Thus, computational methods are the primary alternative. Constant pH Molecular Dynamics (CpHMD) simulation is one of the most accurate p*K*_a_ prediction methods in proteins that contain many charged residues since it captures the coupling between conformational dynamics and residue protonation. Here we study the charge state of a general diffusion porin, OmpF, in which protons exert a crucial regulation of the channel discrimination of small inorganic ions as well as antibiotic translocation. We compare different p*K*_a_ prediction methods, using CpHMD as a benchmark, and discuss the somewhat unusual titration of several acidic residues. The most widely used p*K*_a_ prediction methods, though effective for globular proteins, fall short for membrane-embedded channels either because they were trained using pKa measurements in globular proteins or because of a poor description of the lipidic environment.

## Author summary

Many proteins undergo significant changes in their folding and function depending on the pH of their environment. Therefore, understanding the protonation state of ionizable residues across pH values—often described by their p*K*_a_ —is key to linking structure and function. In the case of channel proteins, their ion selectivity and conductive properties are also affected. However, experimental p*K*_a_ determination is challenging, and computational methods are the primary alternative. Accurate knowledge of the protonation states of ionizable residues is essential for employing powerful molecular simulation tools, even when high-resolution atomic 3D structures of the protein are available. Here, we employ Constant pH Molecular Dynamics (CpHMD) to investigate the pH-dependent protonation behavior of the general diffusion porin OmpF. We compare the predicted p*K*_a_ values of acidic residues with those obtained from other widely used methods, which, though effective for globular proteins, often fail to capture the behavior of membrane-embedded channels. The anomalous behavior of several residues exhibiting large p*K*_a_ shifts is analyzed in detail, providing valuable reference data for simulating the transport of ions, charged metabolites, and antibiotics through the channel.

## Introduction

Electrostatic interactions play a key role in protein structure and function [1,2]. Protein folding and ligand binding as well as many biological processes like transport of ions and small metabolites across cell membranes or enzyme catalysis are tightly regulated by the solution pH. This fact evidences the key role of the protonation or deprotonation of the protein ionizable residues. X-Ray crystallography, Nuclear Magnetic Resonance (NMR) and Cryo-Electron Microscopy have allowed obtaining the 3D structure of an increasing number of proteins at atomic resolution. However, although resolved hydrogens in the crystal structure can be used as a first approximation, this high-resolution structural information is usually supplemented by the pH-dependent charge state of the protein amino acids, often quite different from what would be expected in a free solution because of the protein low dielectric environment and the mutual interaction between ionizable sites. In addition, the local environment of the ionizable residues is highly dynamic and there is a mutual influence between their protonation state and their structural conformation. This is even clearer in protein channels because of the hydrophobic (low polarizability of the membrane) or hydrophilic (higher polarizability in the aqueous pore) environment surrounding the protein residues depending on their location. Also, the net charge and dipole moment of the membrane polar head groups may influence the p*K*_a_ of the residues in their vicinity [3]. These facts have motivated the great effort made in the experimental and theoretical characterization of the ionization equilibria in those sites, that is, in measuring [4,5] or theoretically predicting their p*K*_a_.

There are a relatively large group of protein channels whose ion selectivity, conductance and gating are largely controlled by the electrostatic exclusion due to the interaction between permeating ions and channel ionizable residues, while ion-specific short range non-coulombic interactions play a minor role. This is the case of general diffusion porins and some toxins, which are sometimes included in the family of unconventional channels [6]. Even in non-specific channels the charge state of the protein amino acids is a key factor not only in the channel discrimination of small inorganic ions [7], but also in antibiotic translocation [8] and in their use as analyte biosensing nanodevices [9,10].

**Fig 1:**
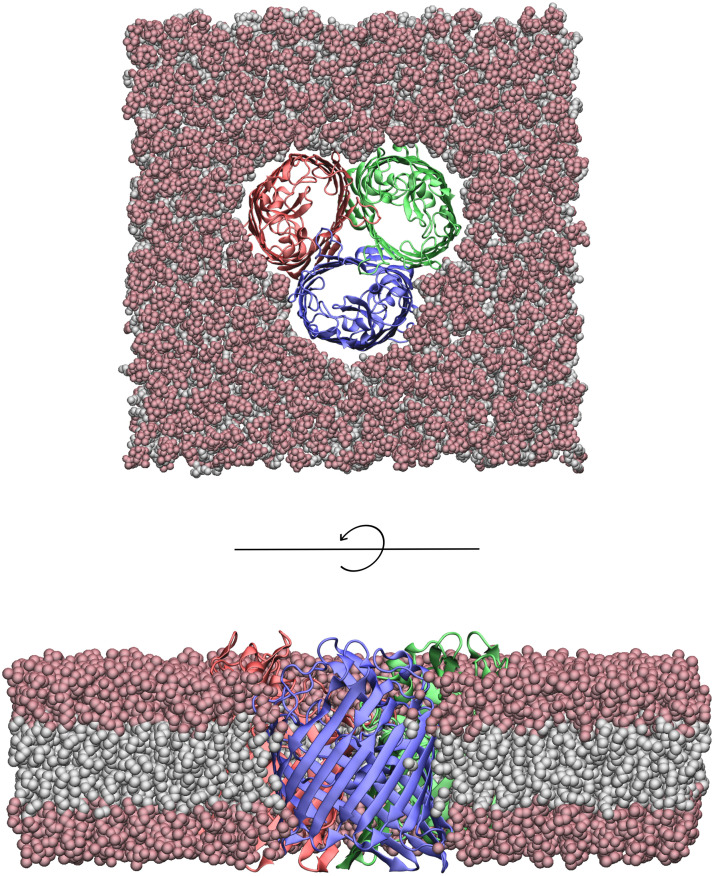
Top and side view of the OmpF trimeric channel embedded in a planar lipid bilayer. Image generated by VMD 2.0 [25].

Here we study the charge state of the outer membrane porin F (OmpF) from *E. coli* (Fig 1). This channel is a major pathway for small hydrophilic molecules through the outer cell wall. The crystal structure of OmpF was resolved more than thirty years ago [11], and there is a large body of experimental characterization (conductance, selectivity, gating, fluctuation analysis, antibiotic permeation, etc.) on the wild-type and mutants [12–24] of this beta-barrel protein.

The importance of this general diffusion porin lies in the fact that it has been identified as one of the key pore-forming proteins involved in antibiotic translocation across the outer membrane of E. coli and other Gram-negative bacteria [8,26,27]. Matching between the charge state of key channel residues and the charge of permeating antibiotic molecules is crucial for antibiotic resistance [28,29]. In addition, modeling and simulation of ion and small solutes transport across this channel requires prior knowledge of the ionization state of amino acid residues and the protein dielectric constant (as well as its value in the aqueous pore and in the membrane). However, the set of local charges and the dielectric constant are not independent from each other. In fact, the dielectric environment regulates the ionization of neighbor residues [30,31]. In absence of experimental measurements of ionization constants of OmpF titratable groups (as happens with most protein channels), several p*K*_a_ prediction methods based on an implicit representation of the protein, the membrane and the solvent and the use of the linearized Poisson-Boltzmann (PB) equation [15,32–34] have been used. Interestingly, all MD simulations of OmpF channel performed over almost twenty years have used nearly the same charge state at neutral pH, despite notable advances in the p*K*_a_ prediction methods [33–38].

All Continuum electrostatics methods employed for p*K*_a_ calculation face the same problem: they need to assume a given value for the dielectric constant of the protein, which is unknown a priori. Many authors have discussed the best assumption of dielectric constant to yield good agreement with experimental results. As pointed out by Varma and Jakobsson [34], this question remains unanswered possibly because of the impossibility of describing the complex dielectric properties of the membrane by a single dielectric constant. Furthermore, the linear addition of electric potentials arising from neighbor charges (an assumption not valid when using the full nonlinear PB solution) is also a challenge of continuum electrostatics methods. Recently, other high-throughput methods for protein p*K*_a_ prediction have become popular which can be broadly catalogued as empirical and MD-based.

Empirical methods, such as PROPKA [39,40] and DeepKa [41,42], estimate p*K*_a_ values based on statistical models that correlate structural features of proteins with known p*K*_a_ values. These methods are popular due to their speed and simplicity, providing reasonably accurate p*K*_a_ estimates for many residues, mainly in relatively stable environments. However, empirical methods tend to overlook the dynamic nature of proteins, particularly in complex environments like membrane channels, where conformational changes and interactions with surrounding water molecules significantly impact protonation states. As a result, empirical methods may overestimate the protonation of residues embedded deep in the protein or within highly dynamic regions, where solvent accessibility and local conformational shifts are crucial.

PROPKA calculates the p*K*_a_ of ionizable residues in a protein using an empirical and physical rule-based approach. It fine-tunes its calculations using a dataset of experimental p*K*_a_ values for various residues in different protein environments. DeepKa uses deep learning to process structural and environmental features to predict the p*K*_a_. While PROPKA uses a dataset of measured p*K*_a_, DeepKa is trained on p*K*_a_ computations. Both methods have the advantage of a fast, often reliable, p*K*_a_ prediction.

Constant pH Molecular Dynamics (CpHMD) offers a more advanced approach by simulating the protonation states of residues in a dynamic, time-dependent manner and according to the conformational environment and user-specified pH [43]. This capability is vital because pH significantly influences electrostatic interactions and, consequently, the conformational ensemble of biological systems [44–46]. Early CpHMD methods primarily relied on *implicit-solvent models or hybrid-solvent schemes*, which, while faster, presented limitations in accuracy and generality, especially for complex systems [44,47]. This spurred the development of more accurate *all-atom explicit-solvent constant pH methods*, which overcome the approximations of continuum models [43].

Recent advancements have significantly enhanced the efficiency and accuracy of all-atom explicit-solvent CpHMD implementations [48–50]. *Amber PME-CpHMD* offers GPU-accelerated all-atom continuous CpHMD with Particle-Mesh Ewald (PME) electrostatics [50]. This implementation handles titration parameters for various residues across CHARMM c22, Amber ff14sb, and ff19sb force fields, achieving a root-mean-square error (RMSE) of 0.76 pH units and a Pearson’s correlation coefficient (r) of 0.80 for pKa shifts in benchmark proteins [50]. Similarly, the *GROMACS CpHMD* implementation, based on λ-dynamics, introduced a linear interpolation of partial charges for long-range electrostatic interactions, which is computationally more efficient than interpolating potential energy functions [48]. This approach allows for nearly the same speed as a standard MD simulation, with only a 30–40% performance drop, and its computational cost does not increase with the number of titratable sites. GROMACS CpHMD has shown good agreement with experimental pKa values, with Pearson’s correlation r of 0.96 and RMSE of 0.49 for cardiotoxin V, and r of 0.90 and RMSE of 0.98 for HEWL [48].

Unlike empirical and PB-based methods, CpHMD allows the protonation states of residues to fluctuate in response to real-time environmental interactions, such as local conformational shifts, water dynamics, and electrostatic changes. This approach is particularly valuable for studying membrane proteins like the OmpF channel, where residues experience varying degrees of exposure to the solvent and interact with other charged residues in a dynamic environment.

Here we use the GROMACS CpHMD implementation to calculate the p*K*_a_ of acidic residues of OmpF channel (aspartates and glutamates) that might be a priori responsible for the changes in the channel charge state upon titration from pH 8 down to pH 1. These p*K*_a_ are compared to those previously reported by a PB-based approach [15], a popular PB-based pKa predictor, H++ [51] and with values obtained from the two abovementioned empirical methods. We discuss the differences in the p*K*_a_ yielded by these five methods and the large p*K*_a_ shifts predicted for certain residues as well as the implications of their titration curves. Some large p*K*_a_ shifts suggest strong interactions between neighbor residues. We also discuss the apparent negative cooperativity that stems from some titration curves that do not follow the Henderson-Hasselbalch (HH) equation. This analysis of the entire titration curve cannot be done with any of the empiric methods, which only yield p*K*_a_ predictions. We show that such apparent negative cooperativity exhibited in a few cases may be the result of microstate heterogeneity of some residues that, at least in one monomer, explore more than one conformation.

## Methods

### System setup

The PDB structure of the OmpF protein 2OMF [11] was used as starting model. The initial system was prepared using the CHARMM-GUI membrane builder [52]. The OmpF protein was embedded in a bilayer of 595 molecules of 1,2-Dipalmitoyl-sn-glycero-3-phosphocholine (DPPC). Both embedding and subsequent solvation in a box of 15.0 × 15.0 × 15.8 nm^3^ were performed using the CHARMM-GUI membrane builder. The CHARMM36-mar2019-cphmd force field [53] and CHARMM TIP3P water model were used for topology generation. Here, cphmd means the inclusion of CpHMD-specific modifications of bonded parameters for the titratable versions of Asp, Glu, His, Arg and Lys. Details on these modifications are described in [54]. The simulation input files were set up using an in-house python script: initially, all Asp, Glu, His, Arg, and Lys residues were made titratable. CpHMD parameters for Asp, Glu, Lys, and His were obtained from [54], while parameters for Arg were obtained from [55]. We ran several test simulations at different pH ranging from 1 to 10 and found that only acidic residues (Asp and Glu) changed their protonation state, while the basic residues remained in their default protonated state during all the procedure. All the remaining simulations were then performed with only acidic residues allowed to change their protonation state, which resulted in an increased computational performance. After the selection of the titratable residues, KCl was added to ensure a net-neutral system at *t* = 0, and to establish an ion concentration of 150 mM. Additionally, 200 buffer particles were added to compensate for charge fluctuations and maintain a net-neutral system at *t* > 0. For more information on the buffer particles, refer to [48,56]. Finally, an in-house python script was used to generate all CpHMD-specific GROMACS input files.

### CpHMD simulations

CpHMD is based on the λ-dynamics technique developed by Brooks and co-workers [57]. A one-dimensional λ-coordinate with ﬁctitious mass m_λ_ was introduced for each titratable site, and the equations of motion for these additional degrees of freedom were integrated along with the Cartesian positions of the atoms [58]. All CpHMD simulations were run using the GROMACS CpHMD beta. This version is based on the 2021 release branch and modified to include the routines required for performing the λ-dynamics calculations. The source code branch is maintained at www.gitlab.com/gromacs-constantph until it has been fully integrated into the main distribution. Energy minimization used the steepest-descent algorithm. Relaxation was initially performed in the NVT ensemble for 250 ps with a time step of 1 fs, using the Berendsen thermostat [59] with a coupling time of 1 ps and a temperature of 300 K. Bond lengths were constrained using the LINCS algorithm [60], and electrostatics were performed using the PME method [61]. Subsequent relaxation and production runs were made in the NPT ensemble using a time step of 2 fs, with pressure kept at 1 bar using the Berendsen barostat [59] (coupling time 5 ps) while gradually releasing restraints on the heavy atoms. Subsequently, three independent runs of 200 ns were performed with the restraints fully released and the CpHMD λ-dynamics activated. We modified the barrier of the residue D312 (in the three monomers) from the default value of 5 kJ·mol^-1^ to a value of 16.5 kJ·mol^-1^ as described elsewhere [48]. Assuming the three OmpF monomers are structurally identical, the averaging of the protonation states is made over a total equivalent time of 1.8 µs. The Molecular Dynamics (MD) simulations were performed with GROMACS on a cluster using 12 assigned Intel(R) Xeon(R) Gold 5220R CPUs together with an NVIDIA Tesla V100-SXM2-32GB GPU. Under these conditions, the simulation speed was approximately ~20 ns/day for a given pH. Since the trajectories extended to ~200 ns per replica, this corresponds to about 10 days of wall-clock time per pH value and replica.

### Analysis

Mean protonation fractions were obtained by averaging the time-averaged protonation fraction values from the three monomers across the three replicas for each system. Protonation fractions were defined as Nproto/(Nproto + Ndeproto), where Nproto and Ndeproto are the number of simulation frames in which the residue was considered protonated (λ < 0.2) or deprotonated (λ > 0.8), respectively. We also determined protonation fractions using a simple averaging over the lambda variable. The differences in all cases between both methods were well below 1%, so that we used the latter for the results presented here. Convergence of protonation-state sampling was evaluated from the time evolution of protonation fractions. As shown for E2 in S6 Fig, averaging over replicas and monomers, the sampling converges within ~10 ns across all pH conditions. Python scripts were used extensively for trajectory analysis, which included representation of titration curves, determination of the p*K*_a_ of each residue, and generation of summary files in spreadsheet files. The p*K*_a_’s were determined by finding the pH at which the protonation fraction was 0.5, by using a linear interpolation between the range of discrete pH values explored in CpHMD simulations. In this work we used pH values ranging from 1 to 8 in increments of a pH unit. For residues with titration curves following sigmoid-like HH equation, we additionally fitted the protonation fraction values to the HH model, using the p*K*_a_ as a fitting parameter. Differences between the p*K*_a_ values from both procedures were not significant (On average less than 0.04 p*K*_a_ units). We used GromacsWrapper [62] for the reading and processing of the resulting xvg files from the gmx cphmd command.

## Results and discussion

### Comparison between p*K*_a_ predictions using different methods

We analyzed the acidic residues of OmpF channel (aspartates and glutamates) that might be relevant to the changes in the channel charge state upon titration from pH 8 down to pH 1. The choice of this pH range obeys to the fact that E. coli can withstand very acidic environments *in vivo*, like that in the extremely acidic stomach (pH range 1.5–3.5) [63,64]. We compare the OmpF p*K*_a_ values reported by Alcaraz et al. [15], who used a PB solver of UHBD (hereafter denoted as PB_A), the predictions provided by the p*K*_a_ predictor H++ [51] (also based on classical continuum electrostatics), the values provided by the empirical p*K*_a_ predictors PROPKA [39,40] and DeepKa [41,42], and those yielded by our CpHMD simulations in 150 mM KCl solutions when the channel is embedded in a neutral DPPC membrane. Additional CpHMD simulations involving the basic residues (arginines and lysines) were also performed, which showed that the charge state of these basic amino acids remains unaltered within this pH range of 1–8.

**Fig 2:**
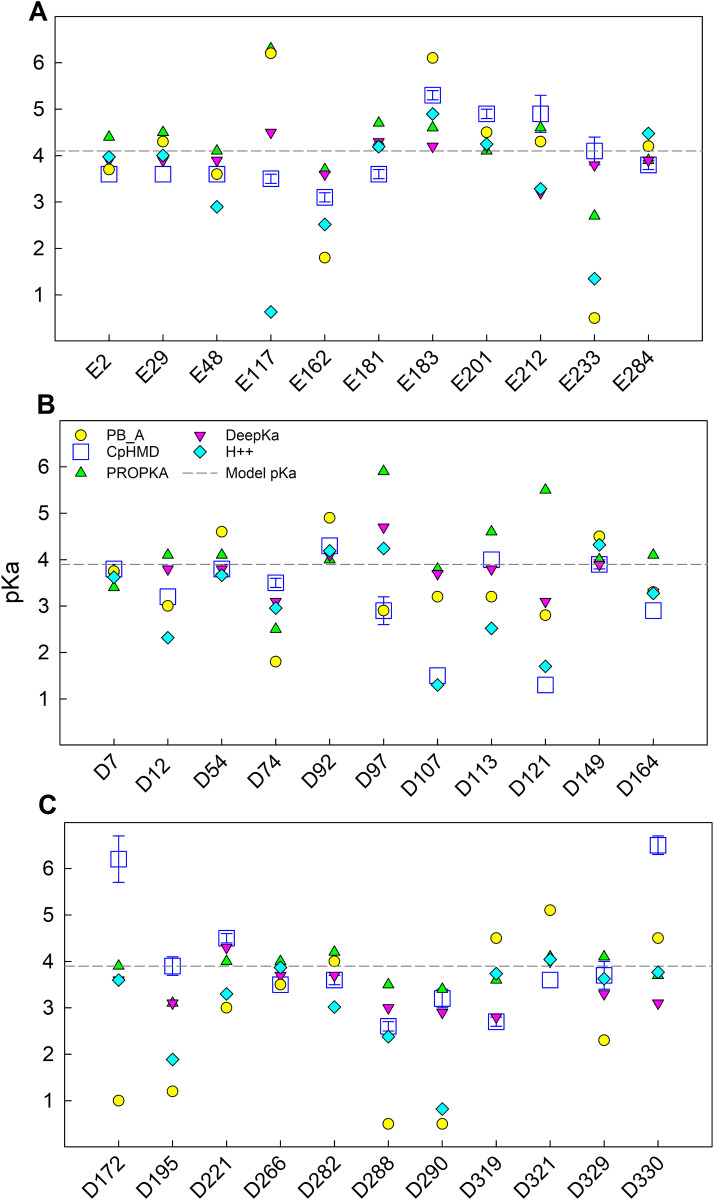
p*K*a prediction of the five analyzed methods for each acidic residue in OmpF. Glutamates are shown in panel **A,** and aspartates in **B** and **C.** Residues with anomalous ionization (p*K*_a_ < 1 or p*K*_a_ > 8) are not included in this figure but shown in Table 2. Model p*K*_a_ is depicted by a dash line.

Fig 2 displays the p*K*_a_ calculated according to the five methods above mentioned, together with the model p*K*_a_ [65] depicted as a reference dash line. Glutamates (Fig 2A) and aspartates (Fig 2B and 2C) are shown separately. There are another 8 residues that are always protonated (p*K*_a_ > 8) or deprotonated (p*K*_a_ < 1) in this pH range according to the CpHMD prediction. These are later analyzed separately and are not included in Fig 2 but shown in Table 2. The actual p*K*_a_ values of all the residues predicted by the five methods are listed in S1 Table.

**Table 2:** p*K*a values of residues with very large p*K*a shifts.

Residue	CpHMD	PROPKA	DeepKa	PB_A	H++
**D 37**	< 1	4.9	3	0.3	2.9
**E 62**	< 1	2.5	2.6	0.4	< 0
**E 71**	< 1	1.1	4.1	0.5	4.6
**D 126**	< 1	3.2	2.5	0.5	< 0
**D 127**	> 8	7.4	5.5	3.8	6.6
**D 256**	> 8	6	3.2	0.6	< 0
**E 296**	> 8	9.6	7.3	8.9	9.4
**D312**	> 8	4.5	6.5	1.5	4.0

The first feature that stands out is that there are positive and negative p*K*_a_ shifts among the predictions of all methods. If the main factor in the deviation of the p*K*_a_ of a residue from its model p*K*_a_ were the low polarizability of its environment, the p*K*_a_ shifts should be positive since, in general, the residues buried in the protein have a higher probability of being protonated than if they were solvent exposed. As can be seen in Fig 2, this trend is not general, which suggests that in many cases the energy of electrostatic interaction with neighboring residues is high enough to compensate for the Born or solvation energy penalty. Actually, two thirds of the p*K*_a_ shifts predicted by our CpHMD simulations are negative.

Second, we observe that for a small number of residues, the p*K*_a_ predictions of the different methods diverge significantly. This is the case for a key residue in the OmpF constriction: E117 (with predicted p*K*_a_ values between 0.6 and 6.3) and other less significant residues in the channel such as E233, D121 and D172.

**Fig 3:**
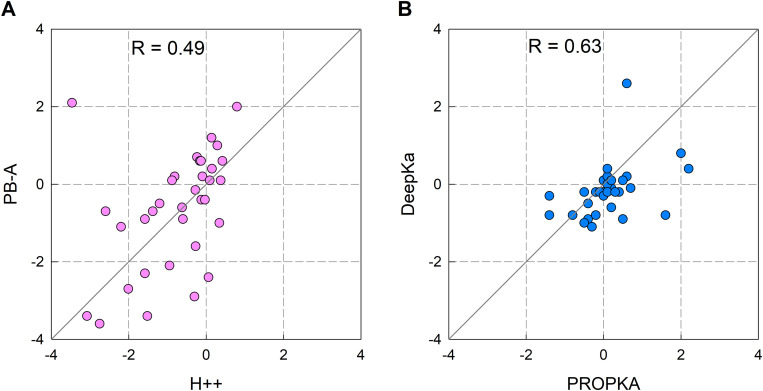
Pairwise comparison of p*K*_a_ shift predictions. p*K*_a_ shift prediction of the two methods based on PB electrostatics (panel **A**) and the two heuristic methods (panel **B**). p*K*_a_ shift means deviation with respect to the model p*K*_a_. Residues with anomalous ionization (p*K*_a_ < 1 or p*K*_a_ > 8) are not included in these plots. *R* is the Pearson correlation coefficient.

The two methods based on PB electrostatics differ considerably in their p*K*_a_ shift prediction (Fig 3A). The Root Mean Square Deviation (RMSD) between them is ca. 1.4 p*K*_a_ units. This might seem surprising, given the similar approach of both methods. However, the evolution from UHBD (1991) to H++ (2005) demonstrates notable advancements in computational methods for p*K*_a_ prediction. UHBD, which solves the PB equation using a finite-difference approach, provides detailed electrostatic potential calculations and global charge distribution analysis but assumes a rigid protein structure, limiting its ability to capture dynamic effects such as conformational changes linked to protonation. In contrast, H++ offers a more flexible approach by incorporating two methods for p*K*_a_ prediction: a clustering algorithm for systems with fewer than 80 ionizable residues, as in the case of OmpF (41 acidic residues per monomer), and Monte Carlo simulations (like traditional PB methods) for larger systems. H++ also introduced side-chain conformational sampling for histidine, glutamine, and asparagine residues, which, while not directly relevant to acidic residues like aspartates and glutamates, could influence their protonation states through neighboring interactions. This ability to model localized conformational flexibility makes H++ better suited than UHBD for capturing protonation-coupled dynamics in protein channels, such as the behavior of acidic residues in the constriction zone of OmpF. Overall, H++’s flexibility, speed, and structural adaptability provide a more accurate and practical solution for p*K*_a_ predictions in dynamic systems like protein channels, where localized and neighboring effects play critical roles.

The comparison of p*K*_a_ shifts predicted by the two heuristic methods, PROPKA and DeepKa (Fig 3B) also reveals some differences between the two methods, although not so large as between the two PB-based methods. The RMSD between them is 0.8 p*K*_a_ units. The key to these differences must be sought in the set of experimental measurements (PROPKA) or protein p*K*_a_ calculations (DeepKa) used in the training of each predictor. In addition, generally both methods predict lower p*K*_a_ shifts than the two PB based predictors.

Table 1 summarizes the performance of PROPKA, DeepKa, PB_A and H++ in the p*K*_a_ prediction of acidic residues (Asp and Glu) by taking CpHMD as benchmark. It displays the overall RMSD of p*K*_a_ from each method with respect to CpHMD calculation, which is reportedly the most reliable p*K*_a_ prediction [41].

**Table 1:** Overall p*K*_a_ RMSD.

p*K*_a_ predictor	RMSD from CpHMD
DeepKa	0.99
PROPKA	1.19
H++	1.32
PB_A	1.70

The comparison between the two empirical predictors, DeepKa and PROPKA, shows that DeepKa outperforms PROPKA in terms of lower RMSD values. DeepKa achieves the lowest RMSD among all methods, aligning with the findings of Wei et al. [66], where DeepKa presented the best results among several p*K*_a_ predictors analyzed. PB_A exhibits the highest RMSD (~1.70), indicating significant deviations from reference (CpHMD) p*K*_a_ values. Its performance is consistent with the challenges of PB-based approaches. H++ demonstrates intermediate performance, with deviation metrics falling between those of PROPKA and PB_A. While not as accurate as DeepKa, it shows a relatively good agreement with CpHMD data.

The observation that DeepKa predictions yield lower RMSD values is not surprising, given that DeepKa was trained using data derived from CpHMD calculations, and thus a better agreement between the two methods could be anticipated. The consideration of CpHMD as the method of better performance, which justifies its selection as a reference in DeepKa, is based on theoretical arguments, as this methodology more accurately represents both the structure and dynamics of the protein along with the various interactions present in the environment of the residues. Additionally, this superiority is also supported by experimental data, where CpHMD has outperformed other methodologies in reproducing experimentally measured p*K*_a_ values [41].

On the other hand, although the overall agreement between CpHMD and DeepKa predicted p*K*_a_ values is not bad (Table 1), significant divergences are observed for certain residues, particularly D256 and to a lesser extent E71, D127 and D330. An analysis of the position of these residues within the protein environment reveals that some of them are located close to the protein-lipid interface. Given that DeepKa was trained and validated using water soluble proteins [41,42], it is expected to yield a poorer prediction for residues that do not meet this condition.

Machine learning p*K*_a_ prediction is rapidly evolving. Less known that DeepKa are two newly developed AI-based p*K*_a_ prediction tools, namely the structure-based KaML-CBT model [67] and the sequence-based KaML-ESM model [68], both reported to achieve higher accuracy than DeepKa. We became aware of these methods when this work was almost finished and compared their p*K*_a_ predictions with those of the other methods (S1 Table and S1 Fig). Our analysis shows that KaML-CBT performs slightly better than DeepKa with respect to CpHMD (RMSD = 0.92), whereas KaML-ESM yields somewhat larger deviations in the overall p*K*_a_ predictions (RMSD = 1.35). Interestingly, despite the improved performance of KaML-CBT in reproducing the p*K*_a_ values for Asp and Glu residues in OmpF, its agreement with CpHMD remains comparable to that of DeepKa for a protein-membrane system like the one studied here. As illustrated in the deviation plots from CpHMD (S2 Fig), both KaML-based methods still show systematic discrepancies relative to CpHMD. This is not unexpected, since their training relied on the new PKAD-3 database [67], which, like the training set used for DeepKa, lacks channel-forming proteins. Consequently, predictions for residues located near the protein–lipid interface, as in the case of OmpF, may be less accurate.

### Residues with anomalous ionization (deprotonated or protonated over the entire pH range 1–8)

**Fig 4:**
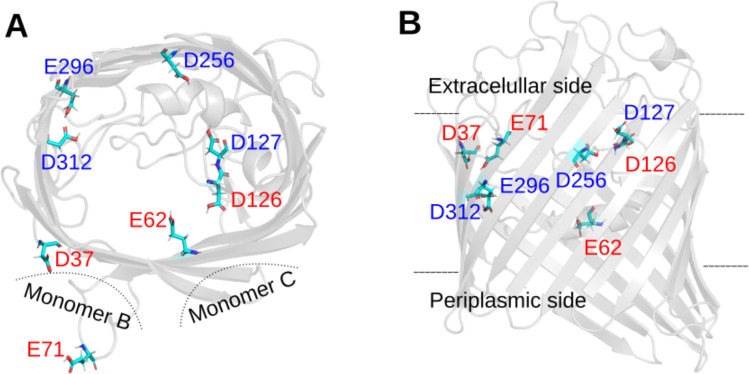
OmpF acidic residues with anomalous p*K*_a_ values. The four residues that remain deprotonated across pH 1-8 are red labeled and the four residues that are protonated in the same pH range are blue labeled. A) Top view of OmpF chain A. B) side view (90° rotation around the x-axis). Dash lines show the relative position of the other monomers (in panel A) and the approximate limits of the membrane (in panel B). Protein renderings were generated using Pymol [69].

Some acidic residues exhibit anomalous ionization according to CpHMD simulations, remaining deprotonated (p*K*_a_ < 1) or protonated (p*K*_a_ > 8) across the pH range studied. D37, E62, E71 and D126 stay charged for the entire pH range, whereas D127, D256, E296 and D312 stay in their neutral form. This prediction contrasts with that of the other four methods explored. The p*K*_a_ obtained using empirical methods show the greatest differences, while methods based on the PB equation agree in some cases with CpHMD (see Table 2 and S1 Table). To find an explanation, we examined the local environment of each residue, including nearby titratable residues, interactions with water molecules, and proximity to the monomer-monomer interface (Fig 4A and 4B).

The local environment of D37 likely favors its deprotonated (charged) state. Although D37 is located at the monomer-monomer interface of the OmpF trimer, this region is still water accessible. Moreover, the interaction with nearby residues such as Thr39 and Tyr98 may favor the deprotonated form through hydrogen bonding or dipolar interactions, which can help accommodate the negative charge by reducing the energetic cost of maintaining a charged carboxylate in this region. Additional hydrogen-bonding interactions mediated by nearby water molecules were also identified with residues Gly67 and Asn161, which may further enhance this effect. If His21 is protonated, its positive charge may contribute to a local electrostatic potential that favors the anionic form of D37 and creates a barrier to proton access, further lowering its p*K*_a_. Even in a neutral state, His21 may still participate in dipolar interactions or hydrogen bonding with D37, which can also contribute to its deprotonation.

The protonation state of E62 is shaped by a network of nearby residues located at the OmpF monomer-monomer interface, including Tyr14, Lys16, Arg42, Lys46, Arg82, and Tyr102. The cluster of basic residues, lysines and arginines, creates a positively charged environment that favors the deprotonated form of E62. As a result, its p*K*_a_ can shift significantly downward compared to its intrinsic value in solution. Although this region is involved in monomer-monomer contacts, it remains accessible to water. The large negative p*K*_a_ shift reflects the dominant influence of these surrounding basic residues.

E71 experiences a weak direct influence from neighboring residues belonging to the same monomer –limited primarily to D74– but due to its position at the monomer-monomer interface (see Fig 4A), this is compensated by a cluster of basic residues from the adjacent monomer, including K80, R82, and R132. Their collective electrostatic influence may favor the deprotonated form of E71, keeping it negatively charged down to pH 1 and below.

D126 is surrounded by a dense cluster of positively charged arginines –R100, R132, R163, R167, and R168– which collectively exert a strong effect favoring its deprotonated form. The nearby titratable residue D127 provides a counteracting effect, but not big enough to compensate for the positive charges. D126 and these arginines are located within the monomer–monomer interface, a relatively restricted region where their side chains are oriented internally facing each other. This close arrangement of positive charges likely creates a strong local electrostatic field that hampers proton association. At the same time, the limited space in this interface may hinder solvent access, making it physically more difficult for a proton to reach D126. As a result, D126 remains deprotonated across the studied pH range.

The protonation state of the aspartate D127 is modulated by nearby acidic residues D126 and D256 as well as basic arginines R132, R167, R168, and R196. Structurally, D127 is in a partially buried region, with low solvent exposure. Interestingly, earlier structural and biochemical studies have highlighted D127 unusual behavior. Karshikoff [32] observed in the OmpF crystal structure that the carboxylate side chain of D127 is positioned within hydrogen-bonding distance (0.26 nm) of the backbone carbonyl of residue A237, a spatial configuration that supports a protonated state. However, subsequent MD simulations by Varma et al. [34] suggested that both protonated and deprotonated forms of D127 are energetically feasible, depending on the local dielectric environment. Experimental work using cysteine-substitution mutants also revealed that D127 is poorly accessible to solvent, implying that the residue is –at least partially– buried [70]. While these earlier findings are not conclusive about D127 charge state under physiological conditions, our CpHMD data support a persistently protonated form, consistent with a buried, hydrogen-bond-stabilized environment.

D256 experiences multiple favorable interactions that support protonation, including contributions from neighboring residues such as D121, Y124, D127, Y231, and E233. The local negative electrostatic potential likely lowers the energetic cost of protonation, thereby favoring the protonated state. Structurally, D256 is in a region near the lipid interface –far from the adjacent monomers– with its side chain oriented internally; all key interacting partners are also in the same monomer.

E296 position in the monomer is like that of D256. It also remains protonated between pH 1 and 8 in our CpHMD simulations, in agreement with earlier computational studies [32,34]. E296 protonation state is influenced by moderate contributions from nearby residues, including E117, D121, Y294, and Y310, and shows particularly strong coupling with D312. All these residues belong to the same monomer. Earlier work by Varma and Jakobsson [33] highlighted the presence of a protonation-coupled network involving E296, D312, Y22, Y310, and E117, where shifts in the protonation state of one residue propagate through the network, altering the charge distribution and potentially the conformational dynamics of the protein. Pongprayoon [71] suggested that full deprotonation of both E296 and D312 significantly increases OmpF flexibility, particularly in the loop L3 region, highlighting the functional sensitivity of the channel to the charge states of these residues. Our CpHMD simulations predict that both E296 and D312 remain neutral from pH 10 down. Under these conditions, we observe no substantial increase in loop L3 flexibility, nor any significant changes in the distances between E296/D312 and E117—the closest residue in loop L3. Pongprayoon [71] also reported that when both residues are charged, the constriction radius decreases. This behavior is not observed in our results when comparing the constriction radius from the CpHMD-derived structure with that from the crystallographic PDB structure. It is worth noting that in Pongprayoon’s study the protonation states of E296 and D312 were manually assigned, without accounting for possible effects on neighboring residues.

### Other residues with large p*K*_a_ shifts: D107 and D121

**Fig 5:**
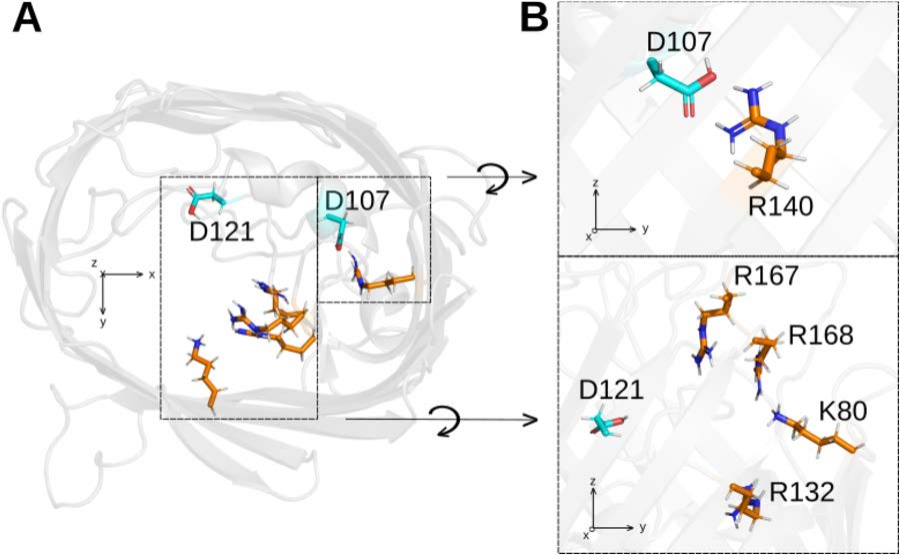
Representation of D107, D121 and the residues supposedly responsible for their large p*K*_a_ shift. Top view in panel **A**, and detailed side view of the neighbor residues in panel **B.** Protein renderings were generated using Pymol [69].

D107 and D121 exhibit considerably larger p*K*_a_ shifts than most acidic residues. Their predicted p*K*_a_ are 1.5 and 1.3, respectively. Both residues are near the OmpF constriction zone (Fig 5A). In fact, D121 is reported by several authors as one of the acidic residues of the constriction, positioned on the loop L3 which is key in the regulation of the channel ion conduction. The simplest explanation of their big negative p*K*_a_ shift is their positively charged environment. D107 is near arginine R140, while D121 is near the group of positive charges, K80, R132, R168 and R167 (Fig 5B). These positive charges might increase the energetic barrier for protons to bind both aspartates, thus decreasing their p*K*_a_’s.

It is worth noting that unlike the transport of small inorganic ions, in which channel constriction plays a predominant role, antibiotic permeation involves other ionizable amino acids from the periplasmic and extracellular vestibules. The study by Ziervogel and Roux [72] shows that two of the residues that we have identified with anomalous ionization (D121 and E62) stabilize the binding of ampicillin and carbenicillin (that contain positively charged NH_3_^+^ moieties) respectively, to OmpF channel. More recently, Acharya et al. [28] also reported that the permeation of charged antibiotics may involve interactions with the L3 loop, particularly with residue D121, further supporting its critical role in antibiotic translocation.

### Residues displaying “slow” titration

Given the number of titratable sites in the OmpF protein close to each other, one would expect a priori that their ionization should be far more complex than that of a collection of independent sites with titration curves following the standard HH sigmoidal shape. For small deviations from HH equation, titration curves are commonly fitted to Hill equation [73], Eq. (1), where *n* is the Hill coefficient, a measure of the cooperativity in a ligand binding process (the protonation of an acidic residue in our case). Then, the fraction *θ* of identical sites that are protonated (or the protonation probability of a single site) is given by


$$\theta ={\left[1+10^{n\left(pKa-pH\right)}\right]}^{-1}$$ (1)

If a residue charge state is not affected by the protonation of its neighbors, its titration curve follows the standard HH equation, i.e., *n* = 1. But, in the opposite case we have two possible scenarios: *n* > 1 or *n* < 1, which represent positive and negative cooperativity, respectively.

**Fig 6:**
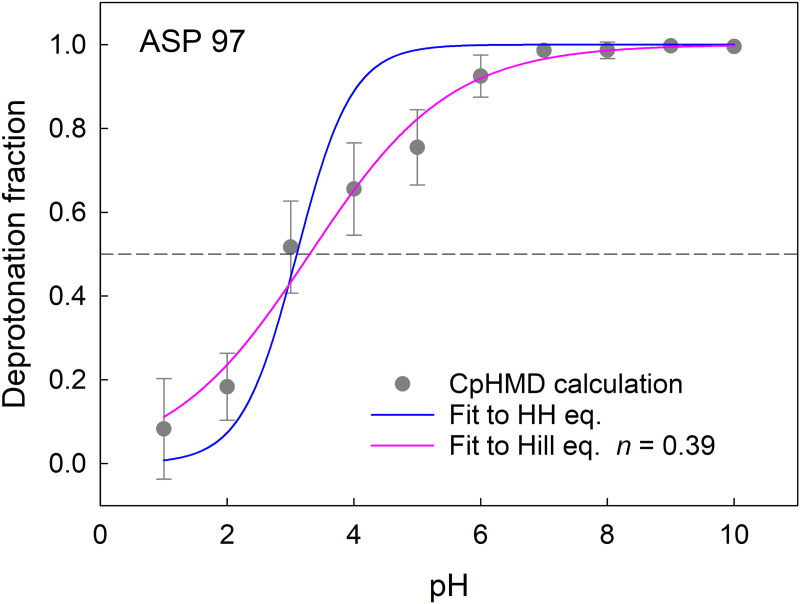
D97 ionization does not follow HH pattern. D97 is an example of OmpF acidic residues that display shallower titration curves. The average fraction of protonation is displayed together with the best fitting curve according to HH equation (blue) and according to Hill equation (pink).

We averaged the charge state of each one of the 41 acidic residues obtained in the three replicas of the three OmpF monomers and fitted them to a) the HH equation to obtain the p*K*_a_ and b) to Hill equation (taking p*K*_a_ and *n* as free parameters). In most cases the two nonlinear fittings yielded virtually the same p*K*_a_ (differences less than 0.1) and values for *n* very close to 1. However, there were a few exceptions in which the average of the 9 protonation curves (3 replicas x 3 monomers) was much better fitted to Hill equation (with *n* values around 0.3-0.4) than to HH equation. Also in these few cases, both HH equation and Hill equation led to virtually the same p*K*_a_. A priori, values of the Hill coefficient lower than 1 are associated to negative cooperativity between neighbor titratable sites, i.e., shallower titration curves, which implies strong interaction between them. However, negative cooperativity could be also the result of heterogeneity of microstates of a given residue [74,75]. There exists the possibility that a residue spends some time in a spatial conformation (characterized by a local minimum of free energy) with a corresponding p*K*_a_ that differs from the most probable conformation p*K*_a_. If that were the case, the process of averaging of all protonation states along the MD trajectory might yield an apparent negative cooperativity without true physical interaction meaning. Fig 6 shows as an example the protonation state of D97 along an extended pH range. As seen, fitting to Hill equation is better than to HH equation. The p*K*_a_ obtained by interpolation (the pH at which *θ* = 0.5) is 3.0, not very different from the best fitting values according to HH or Hill equation (3.1) and (3.3), respectively).

**Fig 7:**
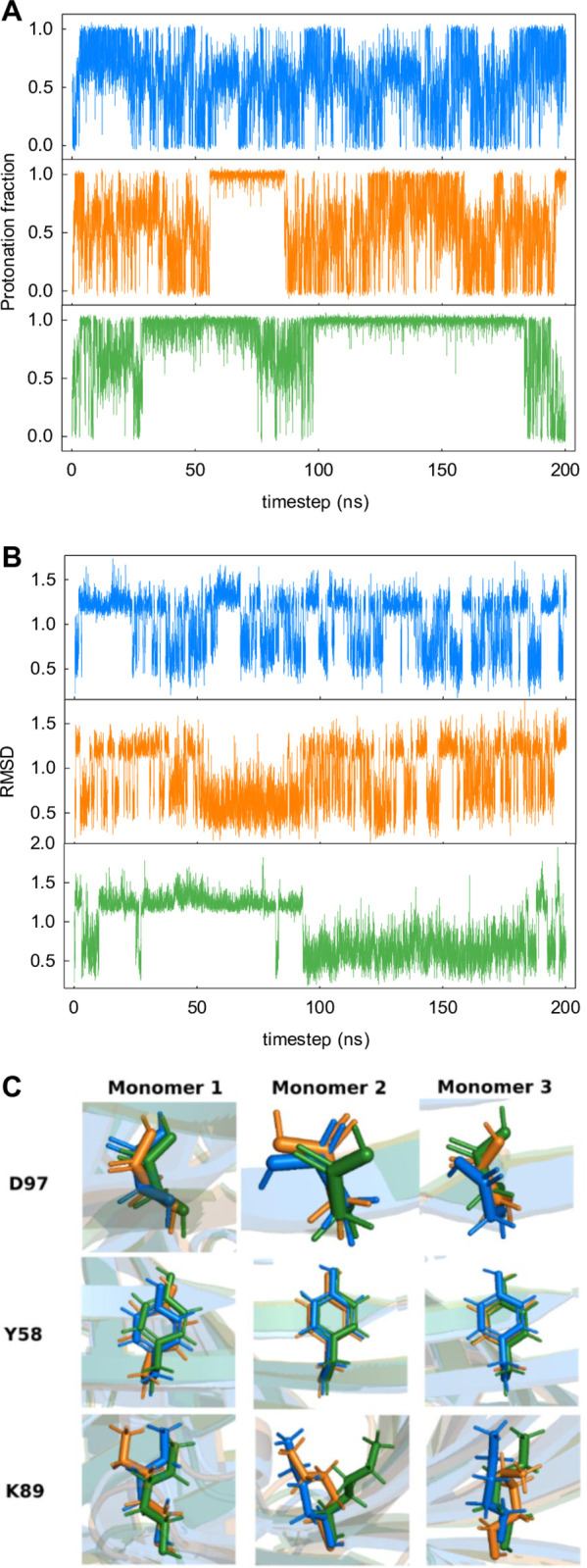
Protonation state and RMSD of D97. Protonation states (A) and RMSD (B) of aspartate D97 in monomer #2 along the simulation time. Representation of D97 side chain and its closest neighbors Y58 and K89 (C). Frame corresponding to *t* = 150 ns. The replicas 1, 2 and 3 are colored blue, orange and green, respectively.

By looking at the protonation states and RMSD (by taking the crystal structure as a reference) of D97 at pH 4 (Fig 7), we see that this residue is accessing different microstates along the simulation time. Incidentally, the information captured by RMSD is also reflected in sidechain dihedral angles, which can likewise be used to monitor the conformational fluctuations of D97 and other residues (S4 Fig). For instance, during a relatively long interval between 100 ns and 175 ns, monomers #1 and #3 display almost no difference between them in the three replicas but in monomer #2 D97 exhibits different protonation states in the three replicas (Fig 7A). In the third replica D97 is protonated all the time (100–175 ns) while in the first and second replicas the residue alternates between its charged and uncharged state (what would be expected for pH 4, not far from the p*K*_a_ 3.3). Interestingly this “unexpected” protonation state seen in the third replica and lasting ca. 80 ns is mirrored in the RMSD of D97 (Fig 7B) within the same time interval. In addition, this “unusual” protonation state in the third simulation replica of monomer #2 might be related to a conformation change of D97 and its closest neighbor residues, Y58 and K89. Fig 7C shows a slight change in the orientation of D97 and K89 in the third replica. Tyrosine Y58 is not titratable and remains with the same orientation in the three replicas for the time interval considered. It is plausible that this and other possible microstates of D97 contribute to the averaging of the simulation trajectory by slowing down the titration curve but with minimal or no effect on the calculated effective p*K*_a_ of the residue.

### OmpF net charge in acidic environments

**Fig 8:**
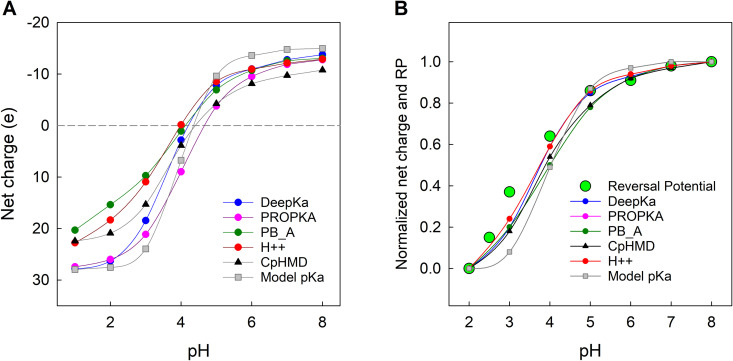
Overall net charge of an OmpF monomer calculated using different p*K*_a_ prediction methods. Differences become most evident under highly acidic conditions. **(A)** Variation of OmpF net charge with pH for each method used to calculate the protonation state of acidic residues. **(B)** Comparison between the measured OmpF reversal potential (RP) in 0.1/1 M KCl [15] (large green symbols) and the net charge predicted by different pKa methods (solid lines as labeled). Both RP and net charge are normalized to their values at pH 2 and pH 8. In both panels the net charge per monomer includes contributions from all other ionizable residues (histidine, arginine and lysine). Solid lines are provided as visual guides.

The differences between the p*K*_a_ predictions obtained from each method become somewhat blurred when evaluating the overall net charge of an OmpF monomer. This stems from the fact that positive and negative p*K*_a_ shifts, depending on the residue, partially compensate each other. Nevertheless, as shown in Fig 8A, clear discrepancies remain in the net charge values, particularly within the pH range 2–4, from which several general trends can be identified. First, all apparent titration curves exhibit slower protonation than predicted using the model p*K*_a_ of aspartate and glutamate residues. Fitting them to the Hill equation yields effective p*K*_a_ values of approximately 3.6-3.9 and Hill coefficients (*n*) of 0.5-0.7. Second, at neutral pH the differences in net charge among methods are minimal. CpHMD predicts a total charge per monomer of –10 *e*, PROPKA –12 *e*, DeepKa –13 *e*, PB_A –13 *e*, and H++ –12 *e*. All these values are slightly less negative than the –15 *e* resulting from the null model (using model p*K*_a_). As illustrated in Fig 8A empiric and PB-based p*K*_a_ predictions overestimate the negative charge of the channel relative to CpHMD. Third, differences in (positive) net charge become more pronounced under highly acidic conditions. For example, at pH 2, CpHMD predicts a positive net charge of +21 *e*, whereas PROPKA and DeepKa give +26 *e*; PB_A + 15 *e* and H++ + 18 *e*. These values are all slightly lower than the + 28 *e* predicted from the null model.

Unfortunately, the net charge of the channel at a given pH, obtained by simply adding up the charges of the ionizable sites, does not have a direct correlation with conductance, RP, or other experimentally accessible observable. This is due, among other reasons, to the fact that the spatial distribution of charges, as well as their position, whether buried or exposed to the solvent, are overlooked. [15,20]. In the particular case of OmpF, other effects must also be considered. First, the solvent exposed residues in the pore constriction have a key role in promoting separate pathways for cations and anions, thus enhancing the ionic conductance. In addition, the competitive binding of cations and protons to D113 and E117 also exerts a substantial influence on the channel permeating properties, and this process is strongly dependent on the solution pH [18,24]. Notwithstanding the above, when the channel selectivity is measured in salts with similar diffusivity of cations and anions, as happens with KCl solutions, the RP should reflect changes in the net charge of the channel when pH titration switches the channel selectivity from cationic to anionic. Fig 8B shows the comparison of OmpF RP measured in 0.1/1 M KCl solutions at several pH [15] superimposed with the change of net charge obtained from different pKa prediction methods across the same pH range. To allow this comparison all datasets have been normalized to their values at pH 2 and pH 8 (the range of experimental data available for RP). There is a common pattern in the pH dependence of the net charge of an OmpF monomer, predicted by different methods, and the measured RP. The fact that all p*K*_a_ prediction methods yield less shallow titration curves than the measured RP in the pH range 3–4 stresses the limitations of using the channel net charge as the only modulator of channel selectivity as explained above.

## Conclusions

Given the difficulty of experimentally measuring the p*K*_a_ of channel proteins, we have resorted to calculating protonation states using one of the most advanced computational tools: the CpHMD method. Its main advantages over other p*K*_a_ predictors for channel proteins can be summarized as follows. First, CpHMD captures the coupling between conformational dynamics and residue protonation states. Second, it circumvents the need to assign *a priori* a dielectric constant to the protein environment: a key limitation of p*K*_a_ predictors based on Poisson–Boltzmann (PB) electrostatics, which only partially address this issue by assigning different polarizabilities to protein regions depending on their solvent exposure or location within the membrane’s hydrophobic core. Finally, the CpHMD method explicitly incorporates the lipid membrane, accounting for its distinct polar and hydrophobic regions.

To date, the CpHMD method has been primarily applied to investigate pH-induced conformational transitions in proton channels. In this work, we analyzed for the first time the pH-dependent charge distribution of a large, multi-ionic trimeric channel (comprising 340 amino acid residues per monomer) embedded in a lipid bilayer. We focused our work on 41 of the 102 ionizable residues per monomer.

Comparison with other empirical methods widely used for globular proteins (such as PROPKA and DeepKa), recent AI-based p*K*_a_ predictors, as well as with approaches based on electrostatic calculations using static 3D structures, reveals significant discrepancies for many of the acidic residues analyzed. For the empirical methods, these differences likely stem from the fact that the correlations and training datasets used in these heuristic approaches are primarily derived from non-transmembrane proteins. Discrepancies with electrostatic methods may result from inadequate representation of the local dielectric environment and its polarizability.

We found that some residues remain either fully protonated or fully deprotonated throughout the entire pH range studied (pH 1–8). Moreover, a substantial fraction of the acidic ionizable residues in OmpF exhibit p*K*_a_ values that deviate significantly from their canonical (model compound) p*K*_a_ values, needing accurate assignment of their protonation states. The calculated p*K*_a_ values can thus serve as a reference for future MD simulations of the channel, aimed at characterizing the transport of both small inorganic ions and antibiotic molecules.

Importantly, it must be considered that charged metabolites traversing the channel may influence nearby residues along their translocation pathway, potentially inducing local p*K*_a_ shifts and altering the protonation states of those residues at a given pH. This dynamic coupling indicates that p*K*_a_ values alone are insufficient to fully describe the system. Therefore, full CpHMD simulations are essential to properly capture the interplay between charged metabolites—such as antibiotics—and the channel environment.

The protonation states of the vast majority of 41 acidic residues of each monomer fit well the HH equation within the pH range 1–8. However, a few residues deviate from this behavior, exhibiting titration curves in which the probability of being charged changes more gradually with pH. For these residues, fitting to the Hill equation yields Hill coefficients around 0.3–0.4. Rather than reflecting negative cooperative interactions between each residue and its neighbors, this shallower behavior likely arises from a superposition of multiple microstates with distinct p*K*_a_ values [74]. Indeed, analysis of the MD trajectories for these residues reveals the presence of alternative equilibrium microstates, distinct from the most probable conformation, but of sufficient persistence to significantly influence the ensemble average across the three 200 ns replicas for each of the three monomers.

Future work is required to explore the impact of ionic screening and membrane electrostatics on the protonation states of OmpF ionizable residues, as well as the contribution of key residues to the conductive and selective properties of the channel.

## Supporting information

S1 Table p*K*a predictions for OmpF acidic residues. p*K*_a_ predictions for 41 selected residues (27 Asp and 14 Glu) obtained with seven different methods: CpHMD, PROPKA, DeepKa, PB_A, H^++^, KaML-CBT and KaML-ESM (see main text). Basic residues remain positively charged across the full pH range (1–8). For CpHMD-derived values, uncertainties were estimated from the standard deviation of pKa values across three independent replicas. Protonation fractions were first averaged over time for each residue, and then across equivalent residues in different monomers, increasing statistical sampling. Three titration curves (one per replica) were obtained, yielding three corresponding p*K*_a_ values.
(XLSX)

S1 Fig Comparison of p*K*_a_ prediction by different methods. p*K*_a_ prediction of the seven analyzed methods (CpHMD, PROPKA, DeepKa, PB_A, H^++^, KaML-CBT and KaML-ESM) for each acidic residue in OmpF. Glutamates are shown in panel A, and aspartates in B and C. Residues with anomalous ionization (p*K*_a_ < 1 or p*K*_a_ > 8) are excluded from the plots but included in S1 Table. Model p*K*_a_ is depicted by a dash line.
(TIF)

S2 Fig p*K*_a_ deviation from CpHMD. Prediction of the two methods based on PB electrostatics (panel A), the two heuristic methods (panel B), the two AI-based methods (panel C), and the AI-based methods compared to DeepKa (panels D and E). Plots include 31 residues (aspartates and glutamates), excluding those with anomalous pKa values (p*K*_a_ < 1 or p*K*_a_ > 8). Pearson correlation coefficient is denoted by *R*.
(TIF)

S3 Fig Titration curves of some acidic residues that depart from the standard HH equation. (TIF)

S4 Fig Sidechain dihedral angle and RMSD of D97. Time evolution of the sidechain dihedral angle (χ) and RMSD of residue D97 in monomer #2. Both traces are highly correlated (R = 0.79–0.88 across replicas), showing that dihedral angles capture the same conformational fluctuations as RMSD. In contrast, pairwise correlations between χ(D97) and χ of neighboring residues Y58 and K89 were negligible (R < 0.05), indicating that D97 dynamics are intrinsic and not driven by adjacent residues.
(TIF)

S5 Fig Protonation state and dihedral angle of D113 and E117. Time evolution of the protonation state (A and C) and dihedral angle (B and D) of two ionizable residues of functional importance in OmpF, D113 (A and B) and E117 (C and D), located close to each other in the constriction region. Nine plots are presented for each residue, corresponding to the three replicas and three monomer per residue in OmpF simulations.
(TIF)

S6 Fig Time series of the protonation fraction for Glutamate 2 (p*K*_a_ = 3.6 ± 0.0) as an example of convergence. The data were plotted every 100th frame, averaging over the three replicas and three OmpF monomers (chains). The pH conditions are given. The plots show that the protonation-state sampling at all pH conditions converge after ∼10 ns.
(TIF)
