# Supplementary figures and images for: Predicting residue ionization of OmpF channel using Constant pH Molecular Dynamics as benchmark
Source: PLoS Comput Biol. 2025 Oct 23;21(10):e1013628. doi: 10.1371/journal.pcbi.1013628 (PMC12578334; doi:10.1371/journal.pcbi.1013628)

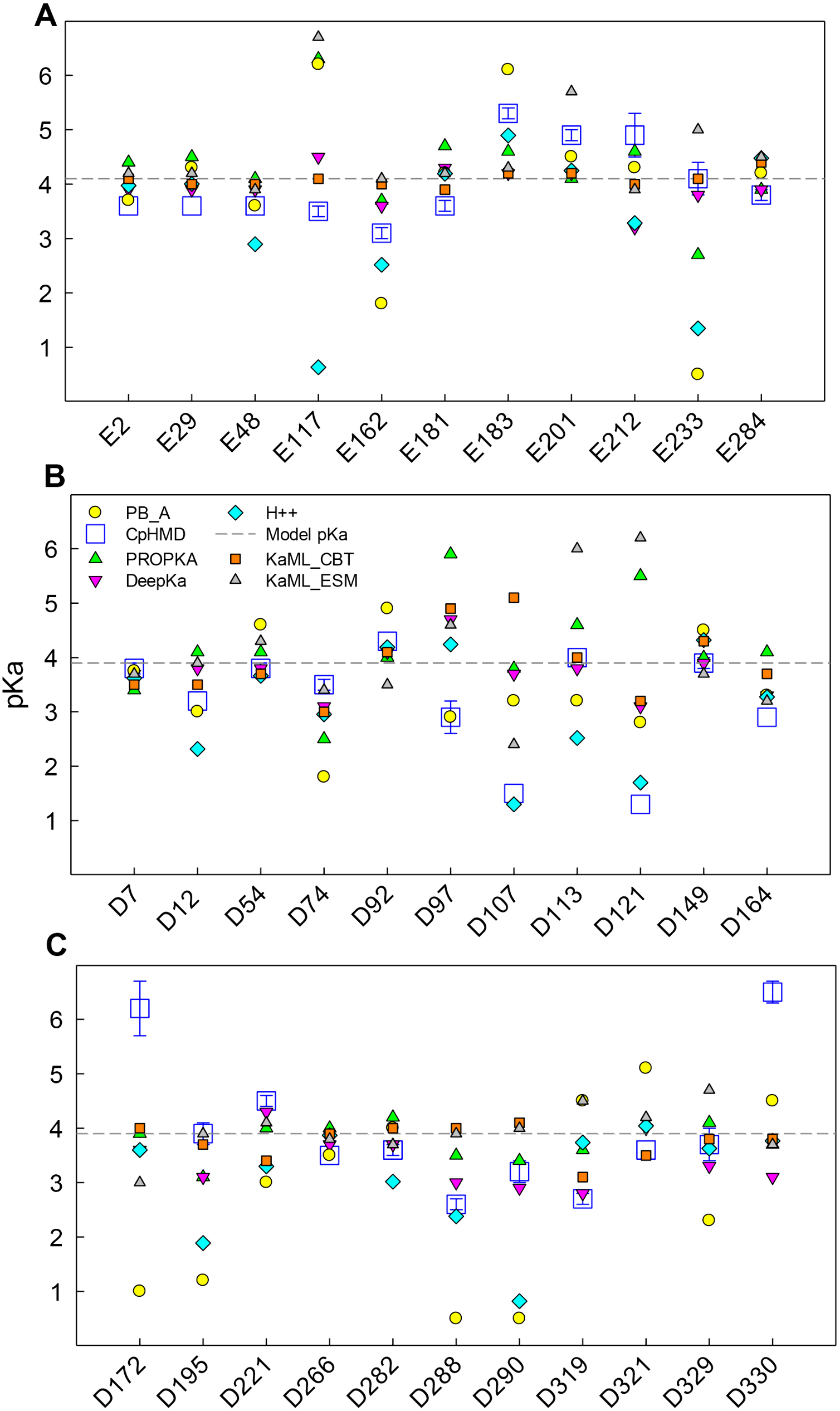

Supplement: S1 Fig — pKa prediction of the seven analyzed methods (CpHMD, PROPKA, DeepKa, PB_A, H++, KaML-CBT and KaML-ESM) for each acidic residue in OmpF. Glutamates are shown in panel A, and aspartates in B and C. Residues with anomalous ionization (pKa < 1 or pKa > 8) are excluded from the plots but included in S1 Table. Model pKa is depicted by a dash line. (TIF) [file pcbi.1013628.s002.tif]

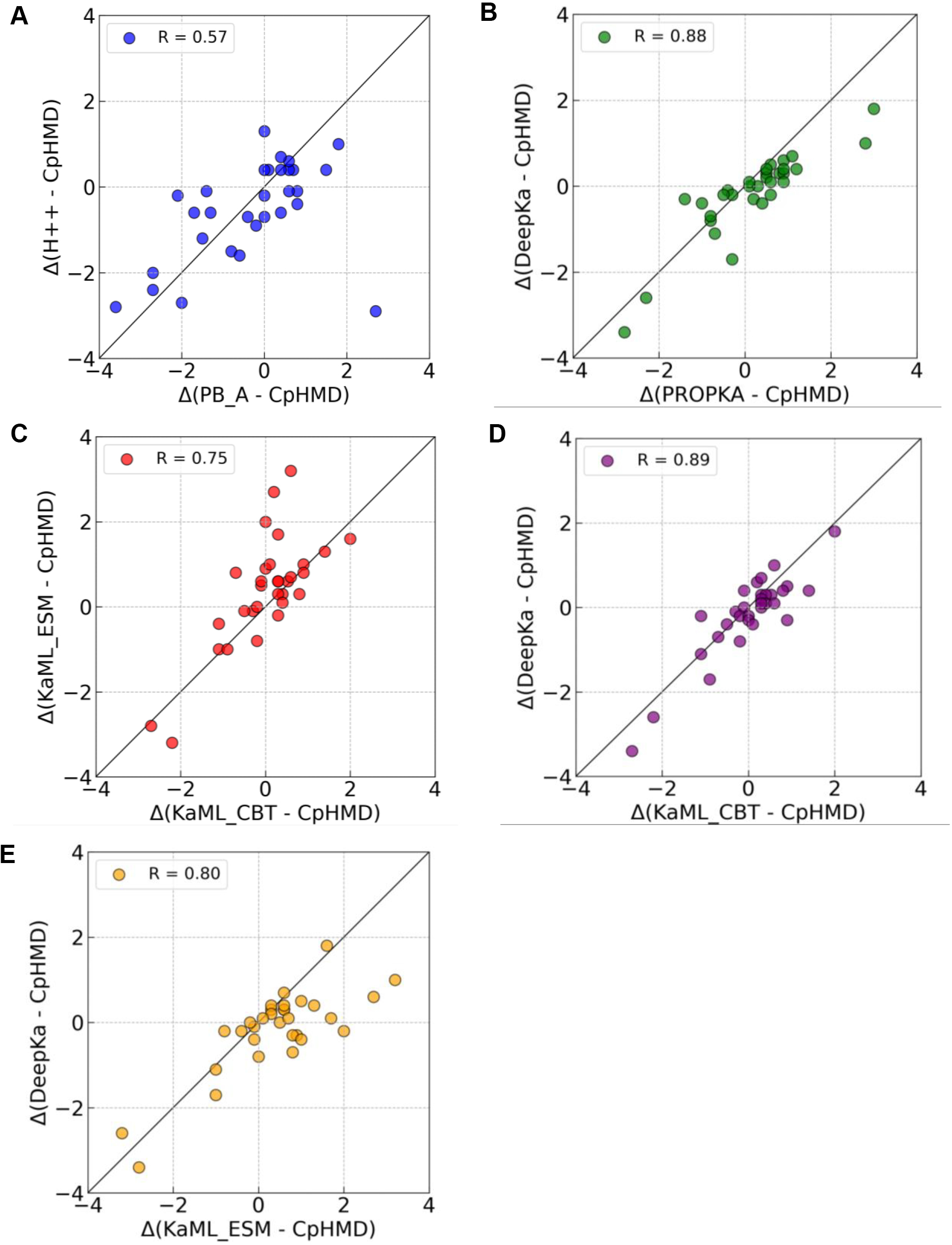

Supplement: S2 Fig — Prediction of the two methods based on PB electrostatics (panel A), the two heuristic methods (panel B), the two AI-based methods (panel C), and the AI-based methods compared to DeepKa (panels D and E). Plots include 31 residues (aspartates and glutamates), excluding those with anomalous pKa values (pKa < 1 or pKa > 8). Pearson correlation coefficient is denoted by R. (TIF) [file pcbi.1013628.s003.tif]

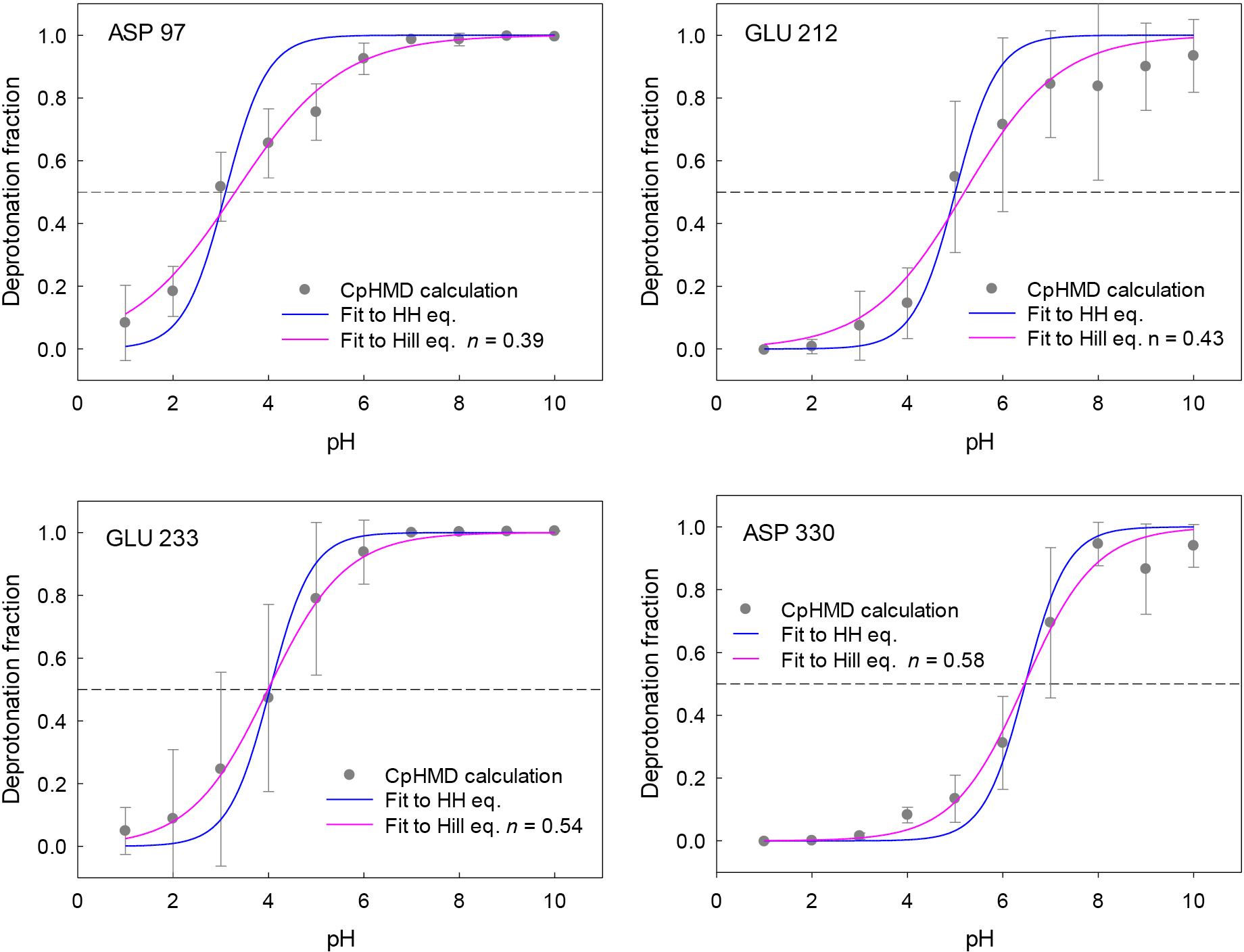

Supplement: S3 Fig — (TIF) [file pcbi.1013628.s004.tif]

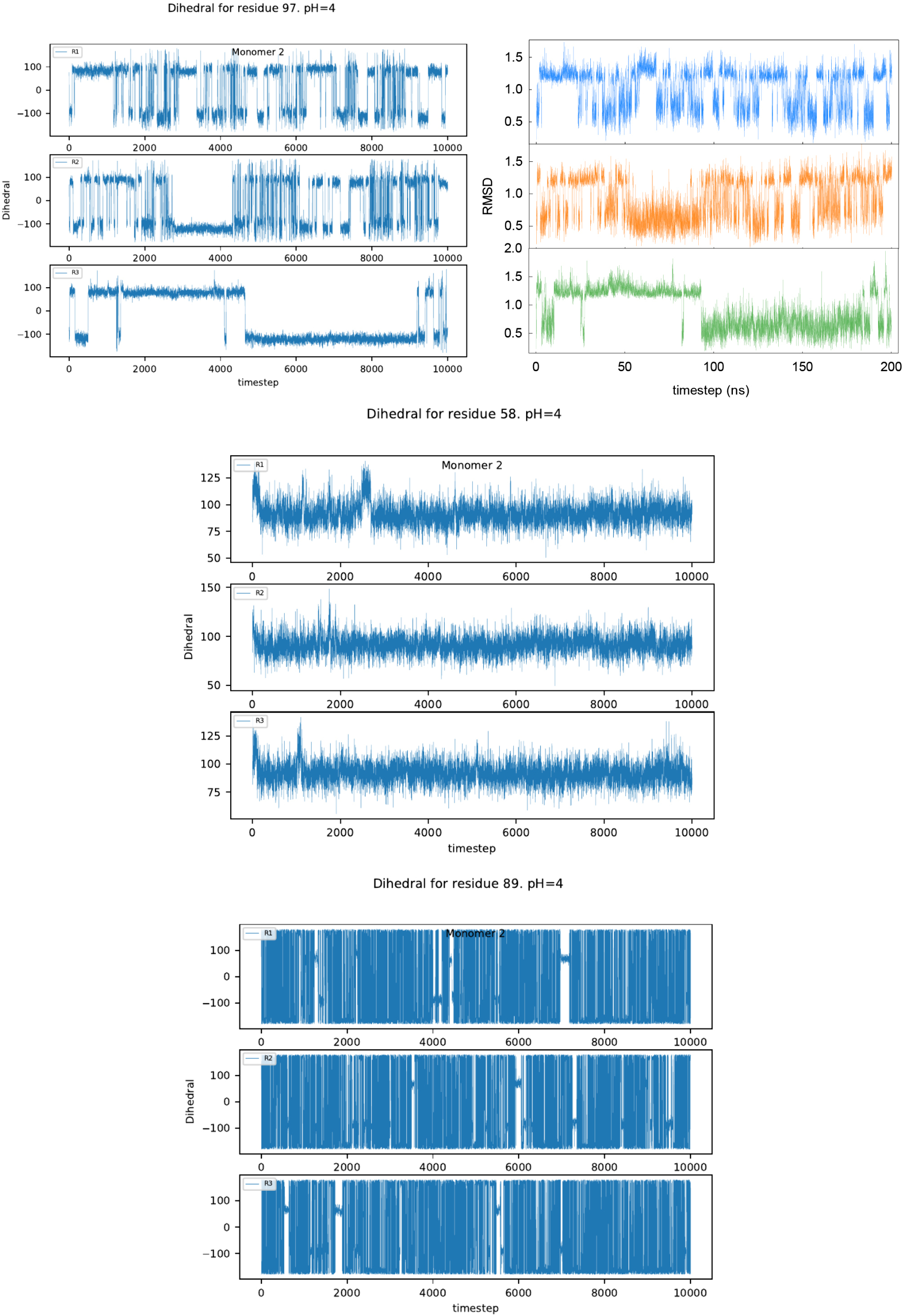

Supplement: S4 Fig — Time evolution of the sidechain dihedral angle (χ) and RMSD of residue D97 in monomer #2. Both traces are highly correlated (R = 0.79–0.88 across replicas), showing that dihedral angles capture the same conformational fluctuations as RMSD. In contrast, pairwise correlations between χ(D97) and χ of neighboring residues Y58 and K89 were negligible (R < 0.05), indicating that D97 dynamics are intrinsic and not driven by adjacent residues. (TIF) [file pcbi.1013628.s005.tif]

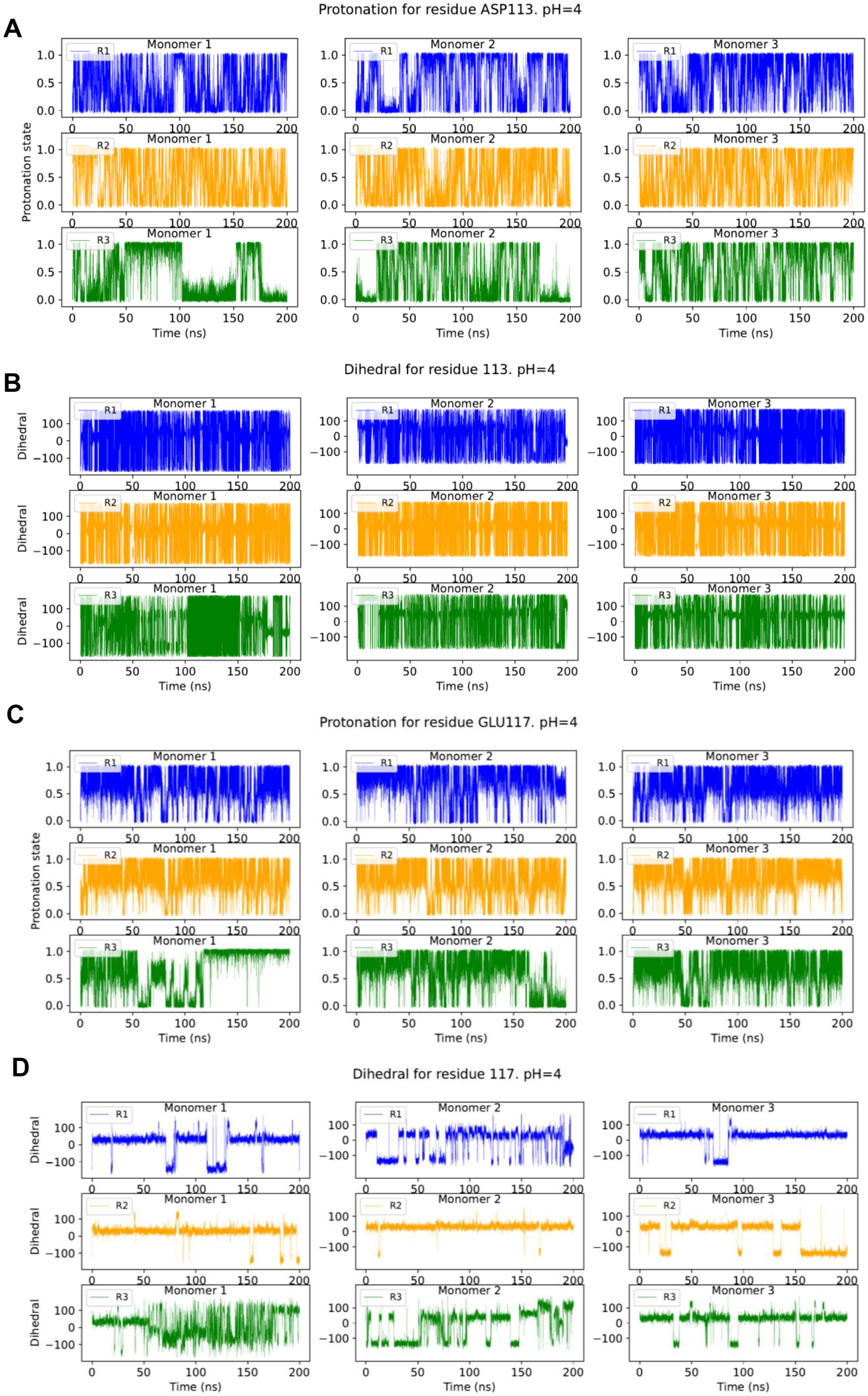

Supplement: S5 Fig — Time evolution of the protonation state (A and C) and dihedral angle (B and D) of two ionizable residues of functional importance in OmpF, D113 (A and B) and E117 (C and D), located close to each other in the constriction region. Nine plots are presented for each residue, corresponding to the three replicas and three monomer per residue in OmpF simulations. (TIF) [file pcbi.1013628.s006.tif]

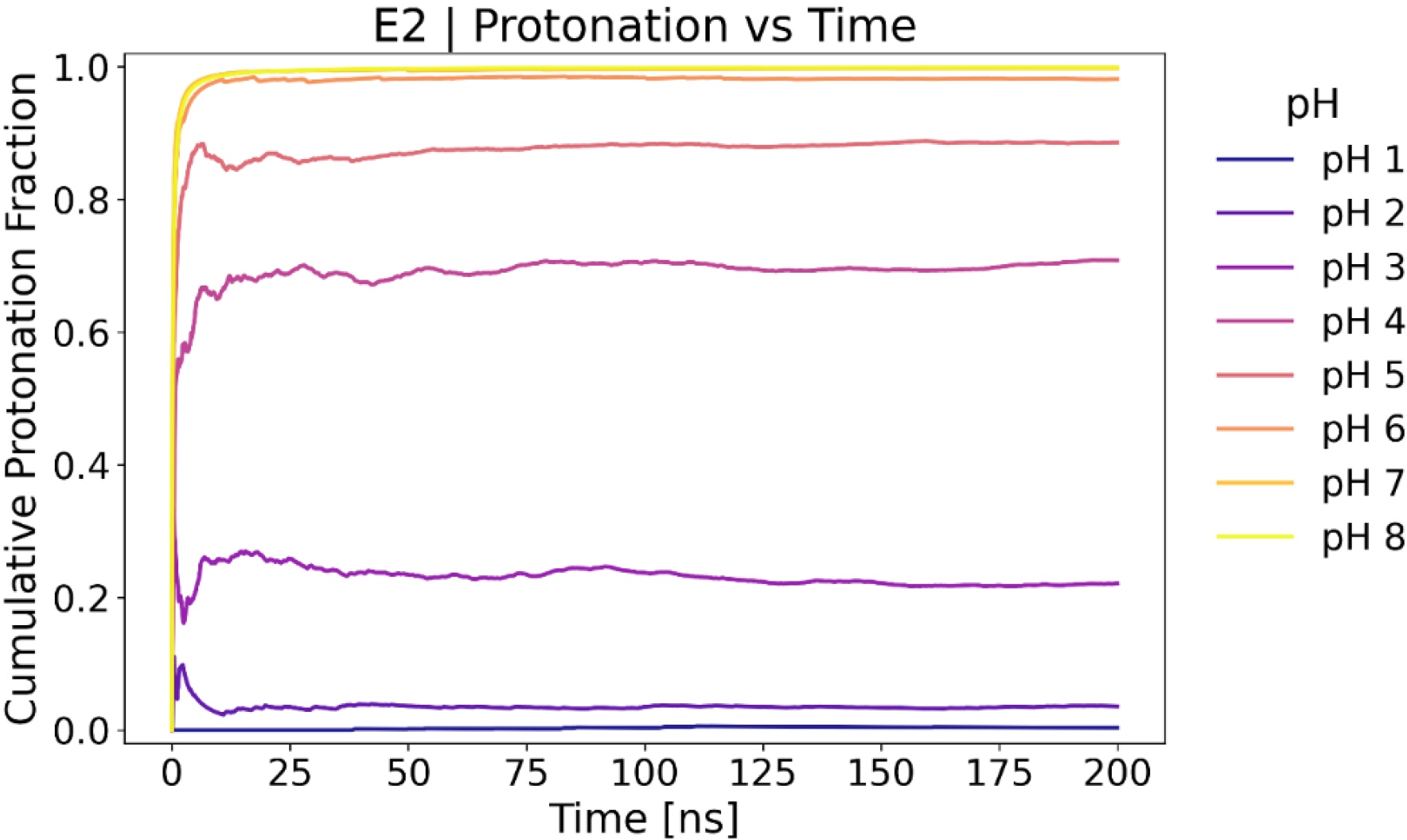

Supplement: S6 Fig — The data were plotted every 100th frame, averaging over the three replicas and three OmpF monomers (chains). The pH conditions are given. The plots show that the protonation-state sampling at all pH conditions converge after ∼10 ns. (TIF) [file pcbi.1013628.s007.tif]
